# The mechanism of social interaction deficits in attention-deficit/hyperactivity disorder children: the role of cognitive flexibility

**DOI:** 10.3389/fpsyt.2025.1691228

**Published:** 2025-11-26

**Authors:** Zicheng Liu, Weizhen Yin, Ziyuan Chen, Wuming He, Zhuoqun Zhang, Sai Sun, Meng Yu

**Affiliations:** 1 Guangdong Provincial Key Laboratory of Development and Education For Special Needs Children, Zhanjiang, Guangdong, China; 2 Guangdong Provincial Key Laboratory of Social Cognitive Neuroscience and Mental Health, Department of Psychology, Sun-Yat Sen University, Guangzhou, China; 3 Guangdong Province Hospital of Chinese People’s Armed Police Forces, Guangzhou, Guangdong, China; 4 The Affiliated Brain Hospital, Guangzhou Medical University, Guangzhou, Guangdong, China; 5 School of Education Science, Lingnan Normal University, Zhanjiang, Guangdong, China; 6 Department of Psychology, School of Public Health, Southern Medical University, Guangzhou, China; 7 Department of Psychiatry, Zhujiang Hospital, Southern Medical University, Guangzhou, China; 8 Frontier Research Institute for Interdisciplinary Sciences, Tohoku University, Sendai, Japan; 9 Research Institute of Electrical Communication, Tohoku University, Sendai, Japan

**Keywords:** ADHD children, cognitive flexibility, model-based strategy, model-free strategy, social interaction deficits

## Abstract

**Background:**

Attention-deficit/hyperactivity disorder (ADHD) is one of the most prevalent mental disorders among children in China. Although the relationship between ADHD symptoms and social interaction deficits has been empirically examined, cognitive flexibility (CF), as a core factor theoretically contributing to ADHD, has still been less studied.

**Aim:**

This study aims to explore how CF influences social interaction deficits using both tradition method (questionnaire surveys) and novel paradigms (Reinforcement learning).

**Method:**

In study 1, we recruited 20 clinically urban Chinese children diagnosed with ADHD (*M* = 9.50, *SD* = 1.82) and 23 control group children *(M* = 9.80, *SD* = 1.86). Questionnaires were used to assess ADHD symptoms, CF, and social interaction deficits (including social self-efficacy and emotion problems). Furthermore, a mediation analysis was conducted. In study 2, 21 urban Chinese children diagnosed with ADHD (*M* = 10.00, *SD* = 2.53) and 21 control children *(M* = 9.19*, SD* = 1.44) completed a two-stage Markov Decision Task to measure their CF.

**Results:**

Study1 showed that CF mediated the relationship between ADHD symptoms and social interaction deficits in children with ADHD. Study 2 demonstrated that the control group employed both model-based and model-free cognitive strategies; however, the ADHD group did not demonstrate either strategy. These findings indicate a significant difference in CF between ADHD and control groups, particularly in their selection of cognitive strategies.

**Conclusion:**

To sum up, the findings suggest that a low level or deficiency in CF may be a key factor contributing to social problems in children with ADHD. Future research directions are further discussed.

## Introduction

Attention deficit/hyperactivity disorder (ADHD) is a common neurodevelopmental disorder during childhood, primarily characterized with attention deficit, hyperactivity, behavioral and impulsive behaviors (1). Individuals diagnosed with ADHD may suffer from learning difficulties, and exhibit more conduct problems, disruptive behavior disorders, or other mental disorders (2). The global age-standardized prevalence of ADHD was estimated to be at 1.13% in 2019 (3), with a similar incidence rate of around 6.4% among Chinese children (4). In addition to cognitive and emotional challenges, ADHD youth often struggle with poor social relationships with peers and family members (5).

Theoretically, Sergeant and colleagues (6) proposed three facets to explain ADHD: top-down control theory, specific cognitive processes theory as well as energetic factors theory, executive function (EF) is regarded as the core concept of the “top-down” processing theory. Research had consistently highlighted EF as a core factor in ADHD symptoms, both theoretically and empirically (7). Among the various components of EF, cognitive flexibility (CF) is particularly crucial, as it helped us to adapt to a changing environment and affected social interactions (8). CF can be conceptually divided into three parts: a) the ability to think flexibly and maintain adaptable choices to achieve a certain goal; b) the willingness to flexibly adapt to the changing environment; c) the confidence to adapt to new environments and challenges (9). Additionally, some researchers had illuminated that deficits in EF such as cognitive flexibility, working memory, were common in adults with ADHD (10). However, researches on CF in ADHD children, especially in Chinese samples, were limited. Therefore, the main aim of the current study was to investigate the role of CF among Chinese children with ADHD.

Questions related to CF and sociability have long attracted the attention of researchers. Martin and Anderson found that CF was positively correlated with the degree of communication skills in normal groups (11). They also found a positive correlation between CF and ratings of friends in social situations, as well as a positive correlation with confidence in demonstrating communication skills in social situations. Bock and colleagues (12) found that CF predicted social comprehension in children aged 7 to 12 years, even after controlling for age, vocabulary, and other executive function factors. More specifically, Curran (13) found that CF might be especially useful for developing social skills and obtaining social support. These clues shed some light on cognitively flexible people might be better at overcoming social barriers and attaining social self-efficacy.

Researches indicated that ADHD children faced various social interaction problems both at home and in school. For example, Ros and Graziano (14) reviewed that the most affected domain of ADHD children’s social functioning was peer interaction. Specifically, Hoza (15) revealed that ADHD children significantly experienced higher rates of peer rejection, with an estimated proportion of 50%-80%. Notably, ADHD symptoms can exacerbate peer relationship problems, leading to greater social maladjustment (16), which can severely impact their self-confidence in social interactions.

Social self-efficacy (SSE) is a key measure of social function as it provided individuals with the confidence to employ necessary behavioral strategies and cognitive resources to meet environmental requirements (17). Erhardt and Hinshaw (18) initially verified the association between the severity of ADHD symptoms and SSE in Western children with ADHD. However, research on SSE among Eastern samples, particularly Chinese children with ADHD, was still scarce due to different social and cultural contexts.

Another important aspect of social interactions is the expression of negative emotions. Spence believed among the factors determining the social response and problems of ADHD patients, cognition and emotion problems were two critical but often overlooked aspects (19). For example, some researchers suggested that ADHD children may possess good social skills but struggle to express their emotions appropriately in specific contexts (20). In addition, Fogleman and colleagues (21) found that the presence of negative emotions can further aggravate social interactions difficulties in children with ADHD. Specifically, heightened negative effect, such as frustration, irritability, or sadness, may impair their ability to regulate emotions, interpret social cues, and respond flexibly to others’ behaviors. These difficulties often lead to misunderstandings, impulsive reactions, and even withdrawal from peer interactions, thereby intensifying social problems.

Previous researches tried to figured out whether CF could mediate the relationship between ADHD symptoms and social interaction deficits or not. For instance, Tseng and Gau (22) revealed that executive function deficit, as a combination of working memory, planning ability, cognitive flexibility, and inhibition ability, could mediate the link between ADHD symptoms and social interaction problems in adolescents. However, their results showed that CF failed to be a significant mediator. They attributed this unexpected finding to the use of general indicators (not specific social micro dimensions) for measuring social problems, which may have biased the results. In line with Tseng and Gau (22), our study 1 aimed to investigate the mediating role of CF between ADHD symptoms and social interaction deficits. However, unlike them, Study 1 focused on two specific social dimensions, using SSE and negative emotions as proxies for social interaction.

To provide a comprehensive assessment of EF in children, we adopted both questionnaires and performance tasks. Previous studies had indicated that rating scales, such as the Behavior Rating Inventory of Executive Function (BRIEF), were sensitive to the changes in EF even when performance-based assessments remain stable and exhibit strong psychometric properties (23). Qian (24) further applied the BRIEF to ADHD clinical samples in China and found significant deficits in all BRIEF factors among ADHD patients compared with control group. These findings not only confirmed the expected EF impairments in ADHD children but also demonstrated the construct and discriminant validity of the BRIEF. In other words, the BRIEF reliably differentiated ADHD children from their typically developing peers in China as well, indicating that questionnaire-based measures provide a solid and valid assessment of EF, including cognitive flexibility, in both clinical and non-clinical child populations.

In addition, to further investigate cognitive inflexibility in ADHD children, in Study 2, we adopted a two-stage decision making task to measure CF. This experimental design aims to enhance our understanding of the behavioral pattern of ADHD children and, how this inflexible pattern might influence their social interaction compared to a control group. The two-stage decision task involves two cognitive strategies: model-based (MB) and model-free (MF). Model-based cognitive strategy involves the use of an internal representation of the environment to prospectively evaluate and compute the value of potential actions. By contrast, habitual control is characterized as a model-free cognitive learning strategy, in which behavior is guided by accumulated reinforcement intendency through trial-and-error learning, without reliance on an explicit mental presentation of the task structure (25, 26). This task has been widely used to assess CF in normal children and adolescents (27), even patients diagnosed with obsessive-compulsive disorder (OCD) (28). Moreover, another study has shown that the two-stage MB and MF task is a more effective measure of CF than the traditional Wisconsin Card Sorting Test (WCST)—a typical task to quantify CF among young adult populations (29). Notably, task-based measures (i.e., the two-stage decision task) also tend to outperform self-report measures (e.g., questionnaires) (30) in assessing CF. In children, inconsistent findings about the use of cognitive strategy were found. Some studies have shown that, only the MF strategy is apparent in developmentally healthy children aged 8 to 12 (27). Conversely, researchers found that children as young as 5 could use MB decision-making strategy (31). Although Natsheh and Shiflett (32) explored reduced MB strategy use in ADHD animal models, scarce studies had investigated the difference in CF among ADHD children using MB and MF models.

To sum up, by using questionnaires, Study 1 revealed significant differences in CF between ADHD and control groups and demonstrated that CF mediated the association between ADHD symptoms and social interaction deficits, particularly for ADHD children. However, Study 1 relied mainly on self-report data, which may not capture the dynamic and behavioral dimensions of CF. To address this limitation, Study 2 was designed to objectively assess CF using a two-stage reinforcement learning task that examines model-based and model-free cognitive strategies. This task allowed us to observe how children adapt their decisions based on environmental feedback, providing a behavioral index of CF. By comparing these learning patterns between ADHD and typically developing children, Study 2 aimed to further elucidate how cognitive inflexibility contributes to the social and emotional difficulties observed in ADHD.

To be specific, we proposed that, in Study 1, for ADHD children, CF would mediate the relationship between ADHD symptoms severity and SSE as well as emotional symptoms, with a different pattern expected in the control group. This expectation was based on the notion that ADHD children typically exhibit greater variability and deficits in EF components, especially CF. In contrast, typically developing children generally possess more stable executive functioning and fewer ADHD-related behavioral disturbances; as a result, the relationship between ADHD symptom severity, CF, and social interaction problems was expected to be weak or non-significant in the control group. Given the limited evidence so far, in Study 2, we only hypothesized that there would be differences in reinforcement learning strategies between the two groups, without a specifying direction.

## Study 1

### Materials and methods

#### Participants

Regarding sample size, the current study followed the criterion proposed by Murphy et al. (33), which recommends a minimum statistical power of 0.50. Based on this standard, the ideal sample size was estimated to be 19 participants. Our study exceeded this requirement, ensuring adequate statistical power for the analyses. A total of 20 clinically Chinese urban children diagnosed with ADHD were recruited, including 15 boys and 5 girls from grade 1 to grade 7, aged from 7 to 13 years old (*M* = 9.500, *SD* = 1.821) through online recruitment methods. All participants were diagnosed ADHD with no other comorbidities by clinical psychiatrists and then underwent semi-structured clinical interviews with psychotherapists at the hospital. The inclusion criteria for the ADHD group included: 1) a primary diagnosis of ADHD; 2) no history of nervous system diseases, current major depressive episodes, severe anxiety disorders, neurodevelopmental disorders, dyslexia, oppositional defiant disorder, autism spectrum disorder, or intellectual disability; 3) an age range of 6 to 15 years old. The exclusion criteria for the control group were: 1) a history of nervous system disease or other psychological disorders; 2) abnormal development; 3) an age outside the range of 6 to 15 years old.

In addition, a total of 23 typically developing Chinese urban children were recruited as the control group through group chat in WeChat (an instant-messaging tool in China), including 7 boys and 16 girls from grade 1 to grade 7, ranging from 7 to 13 years (*M* = 9.800, *SD* = 1.863). All participants from the control group were screened for ADHD symptoms under the supervision of an assessment specialist using the SNAP-IV questionnaire. Individuals scored below 1.8 (34) were retained to ensure the absence of clinically significant ADHD symptoms. We also required that parents report no psychological history for their children before participation in the study. Five participants were excluded from the study, resulting in an attrition rate of 17.86%. After completing all research procedures, a brief psychological test report was provided to both children and parents within two weeks.

After controlling for conduct problems, significant group differences were found in ADHD symptoms (*F* (1,40)_ADHD symptoms_ = 40.185, *p* < 0.001) and CF (*F* (1,40)_CF_ = 4.253, *p* < 0.05), with the ADHD group showing a higher levels of ADHD symptoms (*M* ± *SD*_ADHD_ = 1.658 ± 0.439; *M* ± *SD*_control_ = 0.527 ± 0.313) and lower level of CF (*M* ± *SD*_ADHD_ = 44.6 ± 12.642; *M* ± *SD*_control_ = 51.870 ± 10.015) compared to the control group. As for the emotional symptoms, we also found significant group differences (*F* (1,40)_emotional symptoms_ = 4.298, *p* = 0.045), with the ADHD group showing a higher level of emotional symptoms (*M* ± *SD*_ADHD_ = 3.550 ± 3.203; *M* ± *SD*_control_ = 0.652 ± 0.775). Although the control group showed a higher level of social self-efficacy (*M* ± *SD*_ADHD_ = 84.950 ± 28.692; *M* ± *SD*_control_ = 91.826 ± 16.849), a statistically significant difference was not found (*F* (1,40)_sse_ = 0.008, *p* = 0.927). Moreover, there was no significant gender difference in either the clinical (*F* (1,17) _ADHD symptoms_ = 0.278, *p* = 0.605; *F* (1,17)_CF_ = 1.027, *p* = 0.325; *F* (1,17)_SSE_ = 0.094, *p* = 0.762; *F* (1,17)_emotional symptoms_ = 0.115, *p* = 0.738) or the control group (*F* (1,20) _ADHD symptoms_ = 2.006, *p* = 0.172; *F* (1,20)_CF_ = 0.138, *p* = 0.715; *F* (1,20)_SSE_ = 0.248, *p* = 0.624; *F* (1,120)_emotional symptoms_ = 0.373, *p* = 0.548) even after controlling for conduct problems.

#### Procedure

The current study received ethical approval from the ethics committee of Department of Psychology of Sun Yat-Sen University and the Affiliation Brain Hospital of Guangzhou Medical University. Informed consent was obtained from all participants and their parents, who were also informed of their right to withdraw from the study at any time. For the recruitment of clinical participants, we posted recruiting posters via official channels of the hospital’s ADHD Prevention Center, such as its websites and WeChat groups. All participants were diagnosed by clinical psychiatrists and then underwent semi-structured clinical interviews with psychotherapists at the hospital. The control group comprised 23 children recruited from the community via posters. All participants from the control group were screened for ADHD symptoms (SNAP-IV scored lower than 1.8) (34) and other psychological histories as reported by children’s parents before participating in the study.

Once they met the inclusion criteria and were recruited as participants, all participants completed the questionnaire measurements within one week. Both the children and their parents completed the set of questionnaires. Parents additionally completed the SNAP-IV and the Strengths and Difficulties Questionnaire. All the subjects and parents who volunteered to participate in the study received a small gift and a book as compensation.

### Measurements

#### ADHD symptoms measuring (SNAP-IV)

The version of Swanson, Nolan, and Pelham Rating Scale (SNAP-IV-P) (35) scale was used to measure ADHD symptoms and was completed by parents in this study. The SNAP-IV-P consists of 26 items with three subscales (Inattention, Hyperactivity Impulsiveness, Oppositional Disobedience), each of which was rated on a 4-point scale from 0 (*none at all*) to 3 (*very frequent*). The Cronbach’s alpha coefficient of the SNAP-IV was 0.95, and the Cronbach’s alpha coefficients of the three subscales were 0.90, 0.89, and 0.88 as indicated by a native study in China (36), suggesting a moderate reliability and validity. Notably, the Cronbach’s alpha coefficient of the SNAP-IV-P scale was 0.966 (*N* = 43) in our study.

#### Cognitive flexibility measuring

The cognitive flexibility scale (CFS) (11) was used to measure the level of CF and was finished by participants themselves in this study CFS includes 12 items, and each item was rated with a 6-point Likert rating from 1 (*Very inconsistent*) to 6 (*Very consistent*). The higher the total score was, the higher the CF was. The CFS has been culturally validated in Chinese youth with good psychometric properties (37). In this study, the Cronbach’s alpha coefficient of CFS was 0.823 (*N* = 43).

#### Social self-efficacy measuring

The Perceived Social Self-Efficacy Scale (PSSES) was originally developed to measure adults’ perceived social self-efficacy (38) and was later culturally adapted for Chinese youth, still showing good psychometric properties with a Cronbach’s alpha coefficient of 0.93 (39). More recently, Xiang (40) administrated the PSSES to a sample of 493 Chinese adolescents, finding that the participants clearly understood the scale items and were able to complete the measure appropriately. The scale consists of 25 items with a unidimensional structure. The PSSES uses a 5-point rating scale, ranging from 1(*low efficacy*) to 5 (*high efficacy*), with higher scores indicating greater SSE is. Participants answered the scale by themselves and the internal consistency coefficient in the present study was 0.959 (*N* = 43).

#### Emotional symptoms and conduct problems measuring

The Strengths and Difficulties Questionnaire (SDQ) (41) consists of 25 items rated on a 3-point scale. In this study, the emotional symptoms and conduct problems subscales were used and filled in by participants’ parents. The reliability and validity of the Chinese version of SDQ are adequate (42). In the present study, the Cronbach’s alpha coefficient for the overall SDQ score, emotional symptoms subscale, and conduct problems subscale was 0.910, 0.836, and 0.809, respectively.

### Data analyses

SPSS 25.0 was used for (partial) correlation, ANOVA, and regression analyses. ANOVA was used to examine the group difference of ADHD severity and CF after controlling for conduct problems as a covariate. In addition, the Process Model 4 developed by Hayes was adopted to establish a mediation model following MacKinnon’s four steps guideline (43). In this model, ADHD symptoms were independent variable, CF was viewed as a mediator, and SSE and emotional symptoms were the dependent variables. Due to the small sample size, the Bootstrap method was adopted to test the mediation effect (44). Given the high comorbidity with conduct disorders noted in previous research (45), conduct problems were included as a covariate.

### Results

#### Descriptive statistics

The partial correlation analyses in the clinical group were presented in **Table 1** of **Supplementary Materials**. Conduct problems were controlled as a covariate.

#### Mediation analyses

For the clinical group, ADHD symptoms were significantly and negatively correlated with CF (*r* = -0.572, *p* = 0.011) and SSE (*r* = -0.572, *p* = 0.010) as well as positively associated with emotional symptoms (*r* = 0.522, *p* = 0.022). In addition, CF was found to be positively associated with SSE (*r* = 0.674, *p* = 0.002) and negatively linked with emotional symptoms (*r* = -0.640, *p* = 0.003).

Firstly, we tested the mediation model with SSE as the dependent variable. All variables were standardized prior to testing the mediating effect in the clinical group. As seen in **Table 1** and **Table 2**, ADHD symptoms had a non-significant direct effect on SSE (step 1:*β* = -0.373, *p* = 0.213), with the total effect (*β* = -0.770, *p* = 0.011) at statistically significant level (*R^2^* = 0.38, *F* (2,17) = 5.214, *p* = 0.017). Next we found ADHD symptoms had a significant effect on CF (step 2: *β* = -0.797, *p* = 0.011). CF significantly predicted SSE (step 3; *β* = 0.498, *p* = 0.028). As **Table 2** demonstrated, Bootstrap test results revealed a significant mediating effect, with in indirect effect of -0.397 and a 95% confidence interval (CI) of [-0.908, -0.021], accounting for 51.52% of the total effect. However, in the control group, Bootstrap test results showed a non-significant mediating effect of CF between ADHD symptoms and SSE, with 95% CI of [-0.652, 0.218] (See **Supplementary Table 2**, **3** for more details on mediation effect analyses of the control group).

**Table 1:** Path coefficients of mediation model in the clinical group (SSE as dependent variable).

Variable	CF					SSE				
	*β*	Boot SE	*t*	95%CI		*β*	Boot SE	*t*	95%CI	
				Low	High				Low	High
Constant	0	<-0.001	0	-0.39	0.392	0	0.169	0	-0.353	0.312
Conduct Problem	0.672	0.682	2.424^*^	0.232	1.252	-0.076	0.262	-0.275	-0.553	0.492
ADHD	-0.797	-0.802	-2.872^*^	-1.220	-0.353	-0.373	0.311	-1.294	-1.026	0.220
CF						0.498	0.220	2.408^*^	0.045	0.909
*R^2^*	0.335					0.545				
*F*(df)	4.291^*^_(2,17)_					6.391 ^**^_(3,16)_				

^*^p < 0.05, ^**^p < 0.01.

**Table 2:** Mediating effect analysis of bootstrap results (SSE as dependent variable).

Effect of type	Effect Size	Boot SE	Bootstrap 95%CI		Relative effect percentage
			Low	High	
Total Effect	-0.770	0.247	-1.309	-0.336	100.00%
Direct Effect	-0.373	0.311	-1.026	0.221	48.48%
Indirect Effect	-0.397	0.231	-0.908	-0.021	51.52%

Next, we analyzed the mediation model with emotional symptoms as the dependent variable. For the clinical group, as shown in **Table 3** and **Table 4**, ADHD symptoms had a non-significant direct effect on emotional symptoms (step 1: *β* = 0.310, *p* = 0.321) with the total effect (*β* = 0.696, *p* = 0.022) at statistically significant level (*R^2^* = 0.343, *F* (2,17) = 4.43, *p* = 0.028). Next, we found ADHD symptoms had a significant effect on CF (step 2: *β* = -0.797, *p* = 0.011). CF significantly predicted emotional symptoms (step 3: *β* = -0.485, *p* = 0.040).

**Table 3:** Path coefficients of mediation model in the clinical group (emotional symptoms as dependent variable).

Variable	CF					Emotional symptoms				
	*β*	Boot SE	*t*	95%CI		*β*	Boot SE	*t*	95%CI	
				Low	High				Low	High
Constant	0	0.194	0	-0.383	0.375	0	0.181	0	-0.346	0.354
Conduct Problem	0.672	0.254	2.424^*^	0.246	1.246	0.149	0.313	0.516	-0.541	0.674
ADHD	-0.797	0.220	-2.872^*^	-1.219	-0.344	0.310	0.312	1.023	-0.260	0.969
CF						-0.485	0.175	-2.234^*^	-0.773	-0.086
*R^2^*	0.335					0.499				
*F*(df)	4.291^*^_(2,17)_					5.311 ^**^_(3,16)_				

^*^p < 0.05, ^**^p < 0.01.

**Table 4:** Mediating effect analysis of bootstrap results (emotional symptoms as dependent variable).

Effect of type	Effect Size	Boot SE	Bootstrap 95%CI		Relative effect percentage
			Low	High	
Total Effect	0.696	0.274	0.151	1.249	100.00%
Direct Effect	0.310	0.312	-0.260	0.969	44.48%
Indirect Effect	0.386	0.171	0.061	0.733	55.52%

^*^p < 0.05, ^**^p < 0.01.

As detailed in **Table 4**, the Bootstrap test showed that the mediating effect was significant in the final step, with a mediation effect of 0.386 and a 95% CI of [0.061, 0.733], accounting for 55.52% of the total effect. In the control group, however, results found that the mediating effect of CF on the relationship between ADHD symptoms and emotional symptoms was not significant, with 95% CI [-0.102, 0.399] (See **Supplementary Table 4**, **5** for more details about the results of the control group on mediation effect analyses).

#### Brief discussion

To sum up, by using questionnaires, Study 1 found differences on CF between ADHD children and the control group. Moreover, we used multi-informant measurements and found that CF did mediate the association between ADHD symptoms and social interaction deficits (both significant in SSE and emotional problems) only for ADHD children. Although Study 1 showed a difference in CF between the two groups, the specific manifestation of this difference was not deeply understood and how this difference would affect their social life was not clear. Therefore, we designed Study 2 to further explore how ADHD children’s CF differentiates that of normal children by conducting laboratory experiments.

## Study 2

### Materials and methods

#### Participants

The present study included 21 Chinese urban children diagnosed with ADHD (17 boys, 6 to 15 years, *M* ± *SD*_age_ = 10.00 ± 2.53; *M* ± *SD*_ADHD symptoms_ = 1.415 ± 0.480) from grade 1 to grade 10, and 21 typically developing Chinese urban children aged 7 to 12 (7 boys, *M* ± *SD*_age_ = 9.19 ± 1.44; *M* ± *SD*_ADHD symptoms_ = 0.738 ± 0.350), from grade 1 to grade 7. All participants and their parents volunteered to participate and provided informed consent. The inclusion criteria for the clinical group and the exclusion criteria for the control group were the same as in Study 1. Seven participants were screened out of the experiment due to their withdraw. Upon completion of the experiment, each family received rewards, as in Study 1.

#### Research and experiment procedure

**Figure 1:**
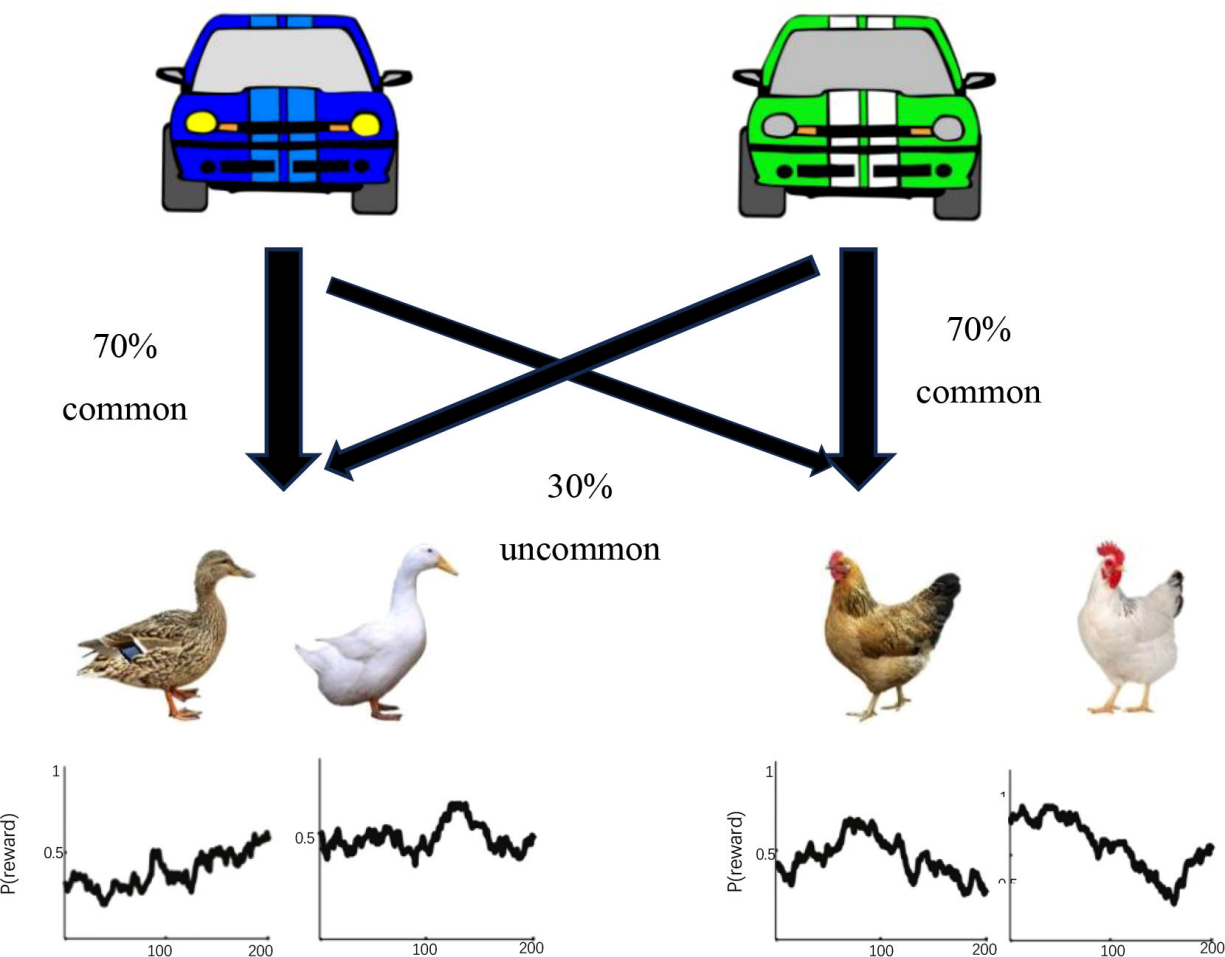
Typical two stages presented to the participants in the experiment.

Informed consent was obtained from both the ADHD children and their parents. The recruitment procedure was the same as in study 1. Participants completed a two-stage Markov decision task (46), designed via E-Prime software, as illustrated in **Figure 1**. In the first stage, the subjects needed to select one of the two options: “blue car” or “green car,” and the choice of either car led to the second stage. In the second stage, participants selected between “white chicken/white duck” or “spotted chicken/spotted duck” option. The outcome, either “laying eggs” (a reward) or “not laying eggs” (no reward), appeared after a choice was made. In this study, participants were instructed that more eggs meant a better outcome.

**Figure 2:**
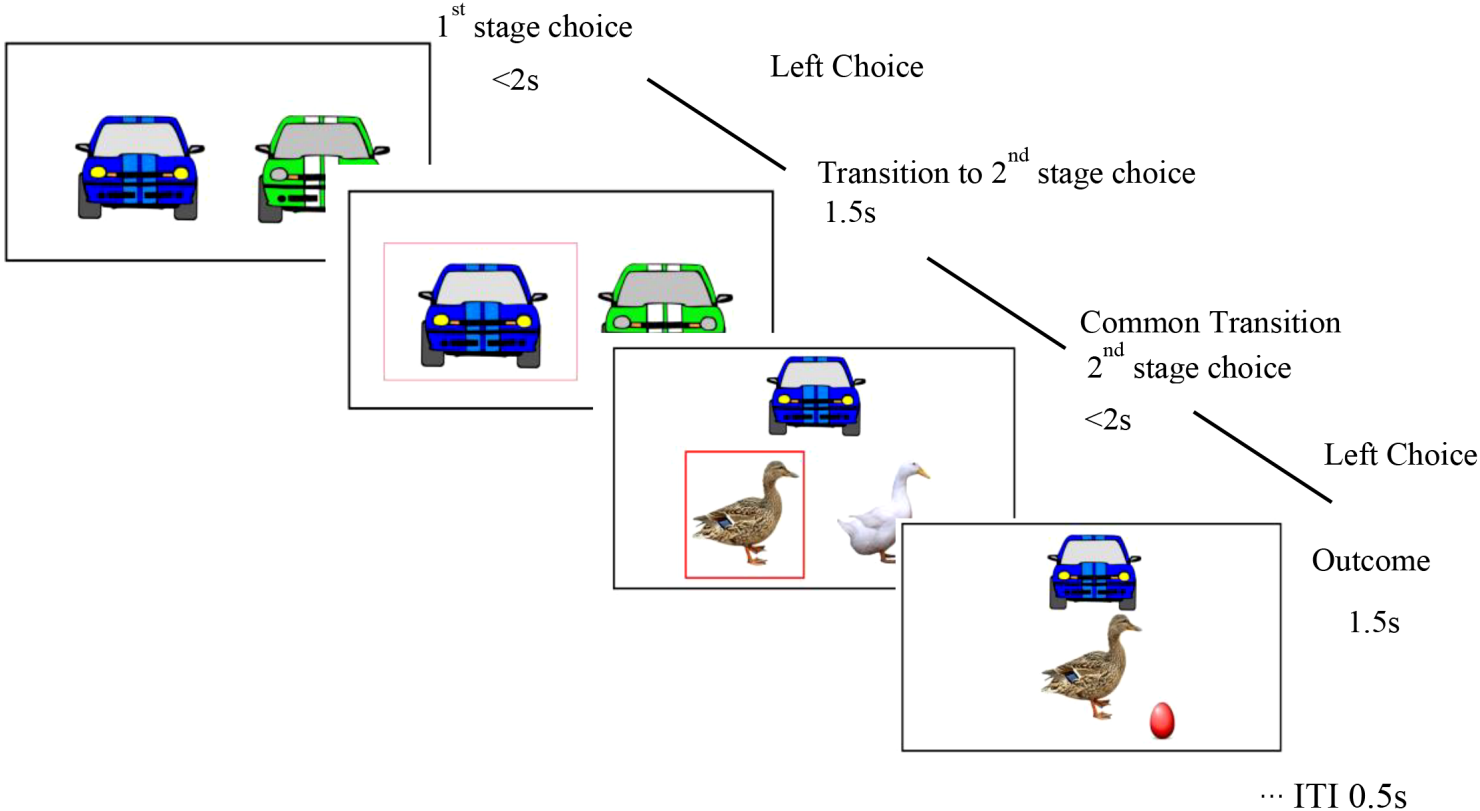
Flow chart of subjects’ selection in the experiment.

In the first stage, participants chose one of the two cars by pressing “1” or “2” within 1.5 seconds. Each car went more frequently to one farm than to the other (70% versus 30%). For example, the blue car had a 70% probability of leading to the duck farm (the common transition) and a 30% probability of leading to the chicken farm (the rare transition), while the green car showed the vice versa. In the second stage, participants selected between two farms (chicken or duck) within 2 seconds to determine if they receive a reward (laying or not laying eggs). The probability of receiving a reward (“laying eggs”) varied slowly and independently over time, changing from 0.250 to 0.750, or vice versa. Therefore, a higher probability in the first stage did not guarantee a higher reward in the second stage given different probability distributions. The entire procedure was demonstrated in **Figure 2**.

Prior to the formal experiment, a detailed instruction was provided to all participants using PowerPoint to familiarize themselves with the task without a time limit. Then, each participant was instructed to complete 200 trials with 50 trials per block. Each trial lasted for 7.5 seconds, and each block lasted for about 6 minutes. Because the experiment program automatically recorded every response and categorized non-selections as incorrect responses, no missing data occurred during the experiment. The entire procedure lasted approximately 25 minutes without breaks.

The internal logic of the method involved predicting different patterns based on reinforcement learning strategies. Specifically, it focused on whether a reward in the second phase of one trial (Trial N) influenced the choice in the first phase of the subsequent trial (Trial N + 1), rather than the second phase of Trial N + 1. Participants using a model-based strategy would adjust their choices based on the internal model of task transitions and expected probabilities after receiving a reward. If a reward followed a rare transition, participants were likely to avoid repeating the same choice in the first stage of the next trial. This forward-looking prediction could also involve a mixture of strategies (31).

### Data analyses

In this study, we primarily focused on behavioral strategies, particularly on how participants adjusted their choices in the next trial (i.e., a first-stage stay) based on the outcome (rewarding or non-rewarding) and transition type (common or rare) of the previous trial. According to the two-stage Markov decision task diagram, these indexes may reflect dynamic cognitive transition processes, including CF and reinforcement learning, which involve updating one’s behavioral strategy in response to changing task contingencies. In other words, participants who can flexibly modify their choices according to trial-by-trial feedback and contextual changes demonstrate higher levels of adaptive decision-making, whereas reduced sensitivity to these variations indicates CF.

A three-way mixed ANOVA was performed with context (common or rare), reward (rewarded or not) and group (ADHD or control) as independent variables, and the percentage of choosing to stay with the same option in the first stage as the dependent variable. In this task, *context* refers to whether the outcome in the second stage followed a common (70%) or rare (30%) transition from the first stage, while *reward* denotes whether participants received a positive feedback (“laying eggs”) in the preceding trial. The *stay percentage* represents the proportion of trials in which participants repeated the same first-stage choice in the next trial. Higher stay percentages following rewarded and common transitions indicate sensitivity to feedback and task structure (i.e., model-based learning), whereas a tendency to repeat choices only after reward regardless of context reflects model-free, habitual responding. These behavioral patterns serve as key indicators of cognitive flexibility and adaptive learning. If the main effect of the reward condition shows significance at the statistically level, it was regarded as a manifestation of the model-free strategy. When there was a significant interaction effect between the reward condition and the transition type, it would demonstrate that the model-based strategy has been used by participants.

Previous studies have indicated that children tend to follow a win-stay/loss-shift (WSLS) strategy, guiding their decisions based on outcome feedback regardless of contextual transitions, this reflects a model-free pattern (27, 47). In contrast, adolescents and adults can use both the probability information from the first stage and reward information from the second stage to update their behaviors, yielding a model-based pattern. In this pattern, participants tend to stay with the same option when the transition is common and the outcome is rewarding, but shift to different option when the transition is rare or the outcome is non-rewarding. In this study, we hypothesized that there could be significant differences in behavioral strategies between the two groups.

### Results

Our results showed a significant interaction between reward and group, *F* (1,40) = 9.045, *η^2p^*= 0.184, *p* = 0.005. Then, a significant three-way interaction among outcome, group, and contexts was found (*F* (1,40) = 4.292, *η^2p^*= 0.097, *p =* 0.045) as well. Next, a simple effects analysis was conducted for the clinical and control group, separately.

**Figure 3:**
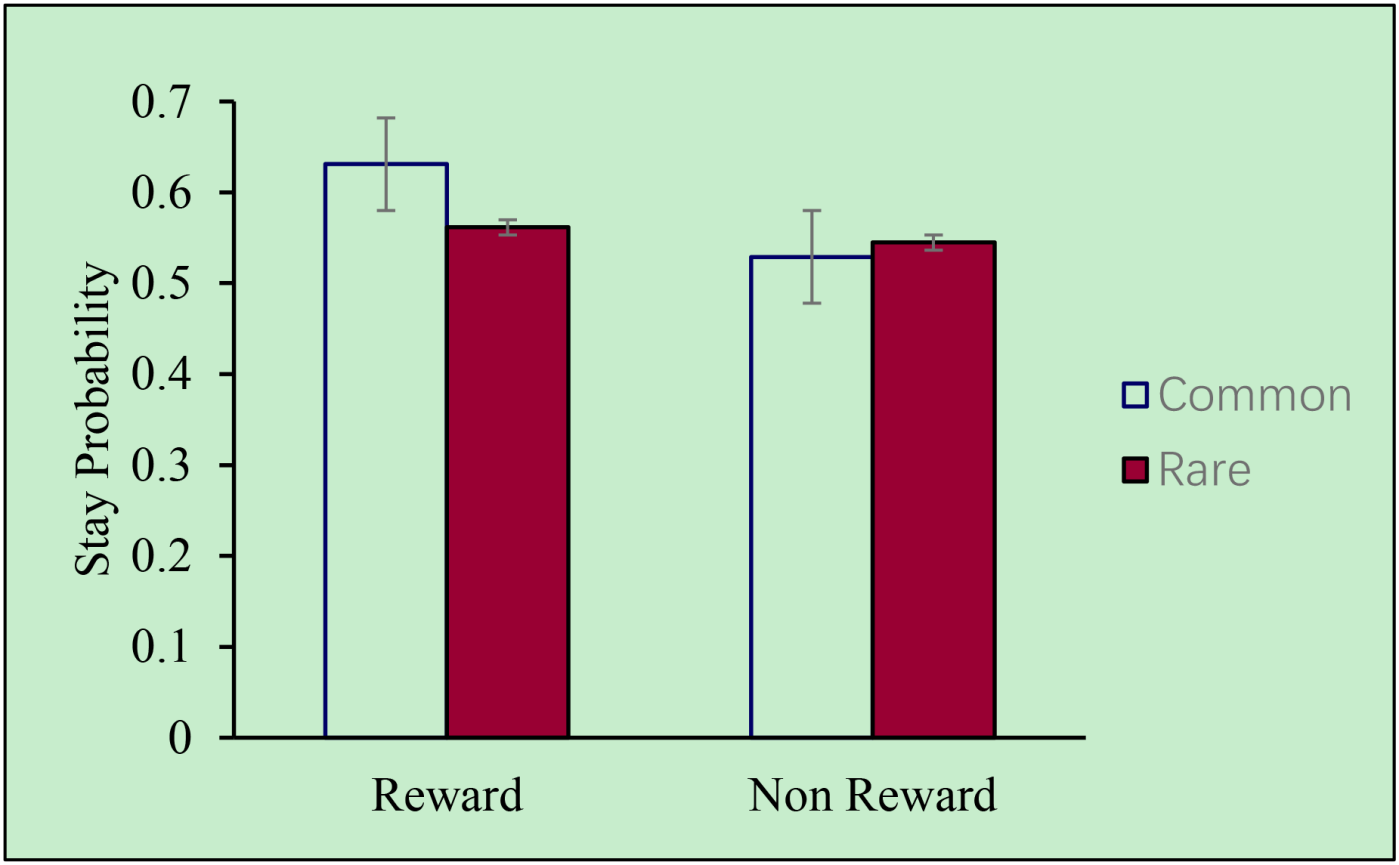
Stay probability of subjects in the control group.

In the control group, as showed in **Figure 3**, there was a main effect of reward (*F* (1,20) = 5.265, *η^2p^* = 0.208, *p* = 0.033), indicating that a model-free strategy was used among typically developing children. This suggests that they tended to use rewarding outcomes to guide their subsequent behaviors while disregarding contextual transitions. Moreover, the interaction between reward and transition type was also significant (*F* (1,20) = 4.401, *η^2p^* = 0.180, *p* = 0.049), suggesting that a model-based cognitive strategy was also utilized. These findings together showed that typically developing children employed both model-based and model-free cognitive learning strategies.

**Figure 4:**
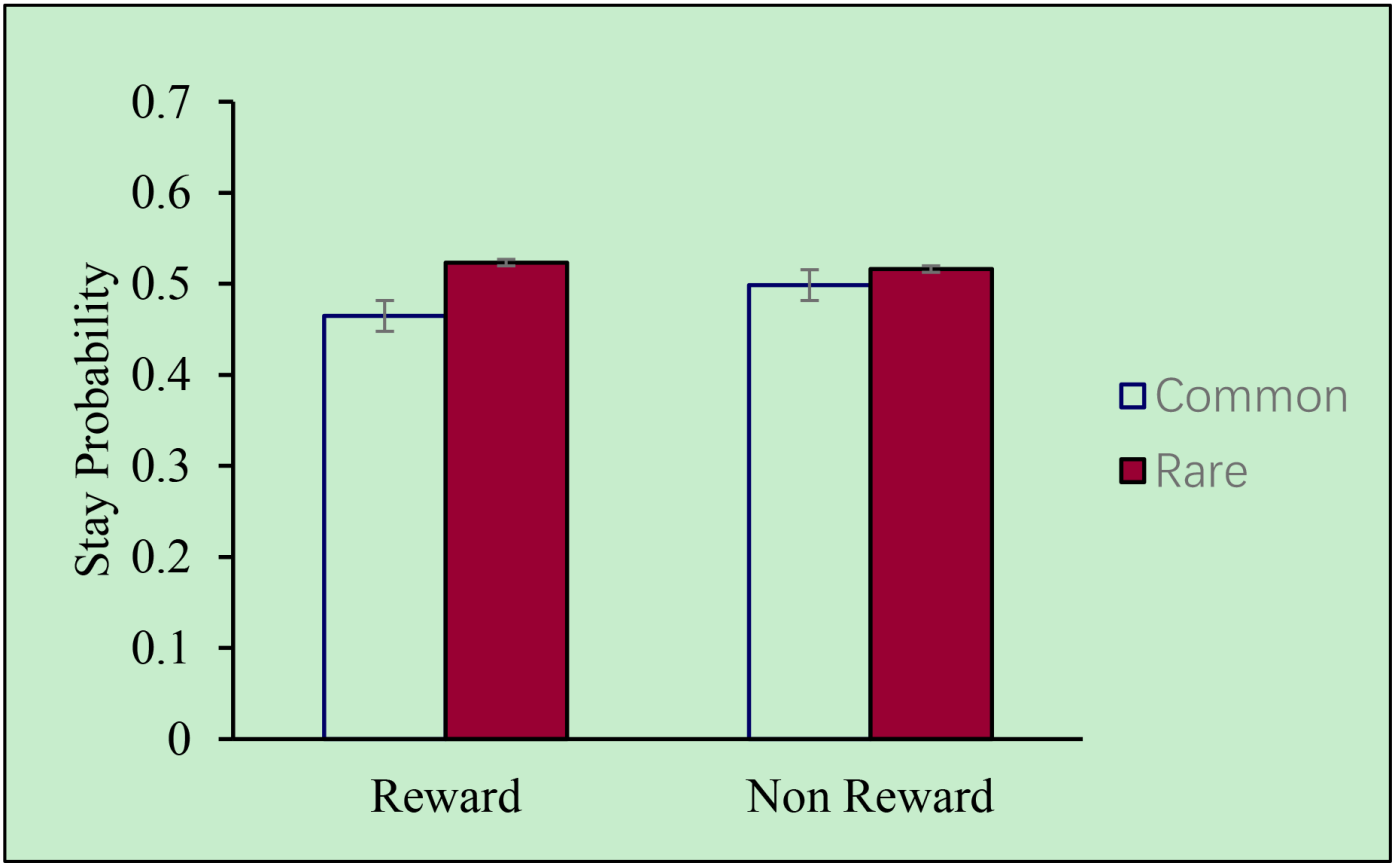
Stay probability of subjects in the clinical group.

However, in the clinical group, as showed in **Figure 4**, no main effect of reward was observed (*F* (1,20) = 3.819, *p* = 0.065), nor was there a significant interaction effect (*F* (1,20) = 0.799, *p* = 0.382). These results indicate a deficit of behavioral flexibility among children with ADHD.

#### Brief discussion

In Study 2, we used a two-stage decision-making paradigm to measure CF in both ADHD children and the control group. We found a hybrid pattern with both model-based and model-free cognitive strategies in the control group, which was consistent with previous research (47). However, neither MB nor MF cognitive strategy was found in ADHD children. This study is the first to investigate CF by conducting a two-stage Markov decision task in Chinese children with ADHD. Our findings replicate those of previous studies (25), and expand the paradigm to ADHD children population.

## Discussion

The current research aimed to investigate the role of CF in ADHD children. In Study 1, we found a significant difference in CF between ADHD children and the control group. Furthermore, using multi-informant measurements, we demonstrated that CF mediated the relationship between ADHD symptoms and SSE, as well as between ADHD symptoms and emotional symptoms. These findings were inconsistent with a previous study (22). In study 2, we adopted a two-stage decision task to specifically analyze the differences in CF between the clinical and control groups. The results showed that the control group employed a mix of MB and MF cognitive reinforcement learning strategies, which were in line with previous researches in both children (47) and adults (25). However, neither a basic MF nor a MB reinforcement learning strategy was identified for ADHD children.

As Daw and colleagues pointed out, MB cognitive strategy would influence individuals’ decision-making, as it allows them to make goal-directed and context-sensitive choices (25). MB learning relies on constructing and continuously updating an internal model of environmental contingencies, namely, how actions lead to outcomes through specific state transitions. This internal model enables individuals to simulate possible consequences of their choices before acting, resulting in more adaptive and sophisticated decision-making compared to the MF strategy, which depends mainly on simple reinforcement of past rewards without considering contextual transitions. Researches further suggested that, for ADHD individuals, the underdevelopment of prefrontal-cortical-related areas, such as, prefrontal-subcortical neurocircuitry (25), abnormal sensitivity to reinforcement and reduced responsiveness to delayed rewards (48), might result in the insufficient use of MB strategy. In adults, Nissan and colleagues (31) found that university students with ADHD exhibited impaired MB strategy use, arguing that ADHD participants faced difficulties in generating complex internal models of task environments compared to typical development peers. Even if ADHD participants could generate internal models, the cognitive effort required to follow these models may pose a challenge. Lastly, they proposed that MB learning could be overwhelmed by the absence of automatic control routines typically provided by the MF system, rendering MB learning less effective in ADHD (31).

From a cognitive perspective, insensitivity to environment stimuli or errors processing during tasks may impair the use of MF cognitive strategy among ADHD children (48). In addition, from a physiological perspective, researchers had found dopamine transfer deficit might account for altered sensitivity to positive reinforcement in children with ADHD (49). The present study contributes to the theoretical understanding of ADHD reinforcement learning by illuminating the deficits in both MB and MF strategies that could exist in clinical children participants, and we may better understand these impacts in the context of social interactions. Furthermore, the lack of MB (and also MF) strategies in children with ADHD may inversely underscore the critical role of CF in peer interactions.

Given that CF in children with ADHD was significantly lower than that in typically developing children, our results further verified the mediating role of CF between ADHD symptom severity and social problems. This can be explained at two levels. First, in rapidly changing social situations, the cognitive mechanism underlying CF help explain why some children with ADHD can maintain relatively normal peer relationships and communicate effectively with their parents and teachers, while others struggle. CF, the ability to switch thoughts or behaviors to adapt to new situations, enables typically developing children to better adapt and integrate into various social contexts. On the contrary, one possible reason for the lack of CF in children with ADHD may be insufficient working memory to process diverse social information, which is crucial in rapidly changing situations (50). Another possible reason was that children with ADHD may ignore important social cues due to a lack of motivation to engage in social communication, eventually leading to a deficiency in CF (51). Future research could explore whether the impact of visuospatial working memory on social interactions in children with ADHD was more significant than that of verbal working memory. In addition, previous studies had shown that planning ability, as an aspect of EF, also influences the social performance of children with ADHD (22). Future research should investigate whether differences in planning ability also exists between ADHD children and typically developing children in China.

It’s worth emphasizing that evaluating CF in ADHD children has important clinical implications for improving the efficacy of therapeutic interventions. Our study provides evidence that CF does *indeed* differentiate between children with ADHD and typically developing children. Given that children’s cognitive abilities were constantly changing, therefore, improving CF in children with ADHD may be more feasible than in adults. Therefore, CF represents a promising target in improving ADHD children’s social interactions in future research.

Last, a novel behavioral measurement of CF was adopted in our design. Specifically, the MB and MF cognitive strategy paradigms in reinforcement learning (25) were used to assess differences in cognitive strategies between ADHD children and the control group. This represented one of the first applications of this paradigm to a clinical sample of children with ADHD. We found the paradigm appropriate for children in terms of both duration and difficulty. Participants also reported enjoying the “game-like” nature of the task and high levels of engagement. Results revealed significant differences in cognitive strategies between ADHD children and the control group, indicating that this paradigm did have discriminative validity. However, only a limited number of studies have applied MB and MF cognitive strategies to examine the underlying physiological and neural mechanisms (52, 53). As the use of MB and MF models expands in physiological research, future research could apply this paradigm to further explore the physiological mechanisms in children with ADHD.

It was also worth mentioning that, given the high reliability of the rating scale results in study 1, our study used empirical research as a counter-evidence to further validate the effectiveness and consistency of rating scale results. The experimental design addressed the issue of limited ecological validity often associated with performance tasks (23) while effectively tested the content validity of both tasks, thereby filling a research gap in this domain.

Several limitations must be acknowledged in the current study. First, there was an imbalance in the ratio of males to females. Although ADHD clinical studies often report a higher prevalence in boys, with ratios of 3:1 or even 5:1 (54), our preliminary analyses indicated no significant gender differences in the variables of interest. However, this imbalance remains a limitation. Second, due to the impact of the pandemic, the present study faced challenges in recruiting participants for both the clinical and control group, resulting in a relatively small sample size. Third, in our study we used a cross-sectional design, which limited our ability to observe changes over time and made it difficult to establish causal relationship between certain characteristics and behavioral conditions. Additionally, the construct of CF examined in this study may not fully encompass all of multidimensional aspects. CF involves not only feedback-based adaptive learning, as measured in the present study, but also other components such as set-shifting, planning, and perspective-taking. Thus, the current results should be interpreted as reflecting one behavioral manifestation of CF rather than the entire construct.

Moreover, although the clinical participants in our study were diagnosed with ADHD through clinical evaluation and did not include other comorbid diagnoses, we still need to pay attention to the potential impact of OC symptoms in future research, especially the well-established overlap in cognitive inflexibility between ADHD and OCD (55, 56), which was not measured and controlled in the current study. Finally, our sample included only school-aged children, while CF and its neural correlates are known to develop dynamically across the lifespan. It is possible that the mechanisms linking CF and social functioning differ between children, adolescents, and adults with ADHD. Therefore, future studies should adopt a developmental and longitudinal perspective, examining how CF evolves over time and how such changes influence social adaptation and emotional regulation in ADHD across different age groups.

## Conclusion

Our research represents a pioneering effort to investigate CF in Chinese children with ADHD using both questionnaires and a novel experimental paradigm. We first explored the mediating role of CF between ADHD symptoms and social interaction deficits in this population, suggesting the important role of CF, especially for ADHD children. In additionally, by using MB and MF paradigm task, the results indicated that children with ADHD significantly underperformed in cognitive tasks compared to their peers, particularly in adaptive adjustments and task switching. These findings highlight the unique challenges faced by children with ADHD in CF and suggest that individualized reinforcement approaches should be considered in intervention strategies to enhance their CF and improve functional performance in daily life. Furthermore, understanding the differences in CF and the developmental trajectory of model-based adaptative learning strategies among typically developing children and those with ADHD can assist with clinical diagnosis, evaluation, and therapeutic inventions, in particular peer interactions.

## Data Availability

The raw data supporting the conclusions of this article will be made available by the authors, without undue reservation.
