# Supplementary material for: The mechanism of social interaction deficits in attention-deficit/hyperactivity disorder children: the role of cognitive flexibility
Source: Front Psychiatry. 2025 Nov 26;16:1691228. doi: 10.3389/fpsyt.2025.1691228 (PMC12690159; doi:10.3389/fpsyt.2025.1691228)
Supplement: Supplementary file 1 [file Table1.docx]

Supplementary materials

**Table 1** Means, standard deviations, and partial correlation coefficients in the clinical group

| Variable | *M* | *SD* | 1 | 2 | 3 | 4 |
| --- | --- | --- | --- | --- | --- | --- |
| 1. ADHD Symptoms | 1.658 | 0.439 | - |  |  |  |
| 2. Cognitive Flexibility | 44.600 | 12.642 | -0.572^*^ | - |  |  |
| 3. Social Self-Efficacy | 84.950 | 28.692 | -0.572^*^ | 0.674^**^ | - |  |
| 4. Emotion Symptoms | 3.550 | 3.203 | 0.522^*^ | -0.640^**^ | -0.481^*^ | - |

*Note*. **p* < 0.05, ***p* < 0.01.

**Table 2** Indirect effect analysis of social self-efficacy in the control group

| Variable | Cognitive Flexibility | | | | | Social Self-efficacy | | | | |
| --- | --- | --- | --- | --- | --- | --- | --- | --- | --- | --- |
|  | *β* | Boot SE | *t* | 95%CI | | *β* | Boot SE | *t* | 95%CI | |
|  |  |  |  | Low | High |  |  |  | Low | High |
| Constant | 0 | 0.209 | 0 | -0.437 | 0.437 | 0 | 0.138 | 0 | -0.288 | 0.288 |
| Conduct Problem | 0.270 | 0.246 | 1.10 | -0.242 | 0.783 | 0.027 | 0.166 | 0.163 | -0.321 | 0.375 |
| ADHD | -0.300 | 0.246 | -1.22 | -0.812 | 0.213 | 0.263 | 0.167 | 1.568 | -0.088 | 0.613 |
| Cognitive Flexibility |  |  |  |  |  | 0.781 | 0.147 | 5.318^*^ | 0.474 | 1.089 |
| *R^2^* | 0.083 | | | | | 0.624 | | | | |
| *F*（df） | 0.910 （2,20） | | | | | 10.508 ^**^ （3,19） | | | | |

**Table 3** Mediating effect analysis of Bootstrap results (social self-efficacy as dependent variable in the control group)

|  |  |  | Bootstrap 95%CI | |
| --- | --- | --- | --- | --- |
| Effect of type | Effect Size | Boot SE | Low | High |
| Total Effect | 0.028 | 0.248 | -0.490 | 0.546 |
| Direct Effect | 0.262 | 0.167 | -0.088 | 0.613 |
| Indirect Effect | -0.234 | 0.217 | -0.652 | 0.218 |

**Table 4** Indirect effect analysis of emotion symptoms in the control group

| Variable | Cognitive Flexibility | | | | | Emotion Symptoms | | | | |
| --- | --- | --- | --- | --- | --- | --- | --- | --- | --- | --- |
|  | *β* | Boot SE | *t* | 95%CI | | *β* | Boot SE | *t* | 95%CI | |
|  |  |  |  | Low | High |  |  |  | Low | High |
| Constant | 0 | 0.209 | 0 | -0.437 | 0.437 | 0 | 0.208 | 0 | -0.435 | 0.435 |
| Conduct Problem | 0.270 | 0.246 | 1.10 | -0.242 | 0.783 | 0.207 | 0.251 | 0.822 | -0.320 | 0.733 |
| ADHD | -0.300 | 0.246 | -1.22 | -0.812 | 0.213 | 0.187 | 0.253 | 0.737 | -0.343 | 0.716 |
| Cognitive Flexibility |  |  |  |  |  | -0.153 | 0.222 | -0.687 | -0.618 | 0.312 |
| *R^2^* | 0.083 | | | | | 0.140 | | | | |
| *F*(df） | 0.910 （2,20） | | | | | 1.035 （3,19） | | | | |

**Table 5** Mediating effect analysis of Bootstrap results (emotion symptoms as dependent variable in the control group)

|  |  |  | Bootstrap 95%CI | |
| --- | --- | --- | --- | --- |
| Effect of type | Effect Size | Boot SE | Low | High |
| Total Effect | 0.232 | 0.241 | -0.270 | 0.735 |
| Direct Effect | 0.186 | 0.253 | -0.343 | 0.716 |
| Indirect Effect | 0.046 | 0.128 | -0.102 | 0.399 |
